# Hypertensive emergency with vasogenic cerebral edema following droxidopa initiation in pure autonomic failure

**DOI:** 10.3389/fneur.2026.1848250

**Published:** 2026-08-20

**Authors:** Min-Ku Kim, Sung-Sik Kim, Jong-Gyu Baek

**Affiliations:** 1 Department of Neurology, Kyung-il Neurology Medicine Center, Daegu, Republic of Korea; 2 Department of Neurology, Daegu Goodmorning Hospital, Daegu, Republic of Korea; 3 Department of Neurology, Yeungnam University College of Medicine, Daegu, Republic of Korea

**Keywords:** dizziness, droxidopa, hypertensive emergency, neurogenic orthostatic hypotension, pure autonomic failure, vasogenic cerebral edema

## Abstract

Pure autonomic failure (PAF) is characterized by neurogenic orthostatic hypotension and marked blood pressure (BP) lability. Droxidopa is widely used for symptomatic management and is generally well tolerated. We report a 60-year-old woman with advanced PAF who developed a hypertensive emergency with vasogenic cerebral edema shortly after initiation of low-dose droxidopa. This case highlights that severe hypertensive complications with reversible cerebral edema may occur even during low-dose droxidopa initiation in vulnerable patients with advanced autonomic dysfunction, underscoring the need for cautious dose titration and close BP monitoring.

## Introduction

Pure autonomic failure (PAF) is a rare, sporadic neurodegenerative *α*-synucleinopathy of the autonomic nervous system, pathologically characterized by predominant peripheral deposition of *α*-synuclein (1). Clinically, it is commonly associated with neurogenic orthostatic hypotension (OH), supine hypertension, and marked blood pressure (BP) lability (1). Droxidopa, a norepinephrine prodrug, is a widely used pharmacologic treatment for the management of neurogenic OH (2, 3). Although hypertension and headache have been reported in association with droxidopa, severe hypertensive emergency complicated by reversible vasogenic cerebral edema following initiation of low-dose treatment appears to be extremely rare (2, 4, 5). Herein, we describe a patient with advanced PAF who developed a hypertensive emergency with reversible vasogenic cerebral edema temporally associated with initiation of low-dose droxidopa.

## Case presentation

A 60-year-old woman presented with a 10-year history of orthostatic dizziness and chronic constipation with recurrent episodes of severe abdominal pain and vomiting of unclear etiology. She had no prior diagnosis of chronic essential hypertension or diabetes mellitus and denied any family history of neurological disorders. Secondary causes of autonomic neuropathy, including amyloidosis, were excluded based on clinical history and laboratory evaluation. She was taking escitalopram 5 mg for mild anxiety and persistent postural perceptual dizziness and these symptoms remained stable. She was not taking any other medications with norepinephrine reuptake–inhibitory activity, including serotonin–norepinephrine reuptake inhibitors. Several years earlier, autonomic function testing demonstrated severe generalized autonomic failure (Composite Autonomic Scoring Scale score 9/10) with reduced postganglionic sudomotor function, impaired cardiovagal responses, and neurogenic OH. Midodrine therapy was initiated, but because there was no marked improvement, follow-up was discontinued.

**Figure 1:**
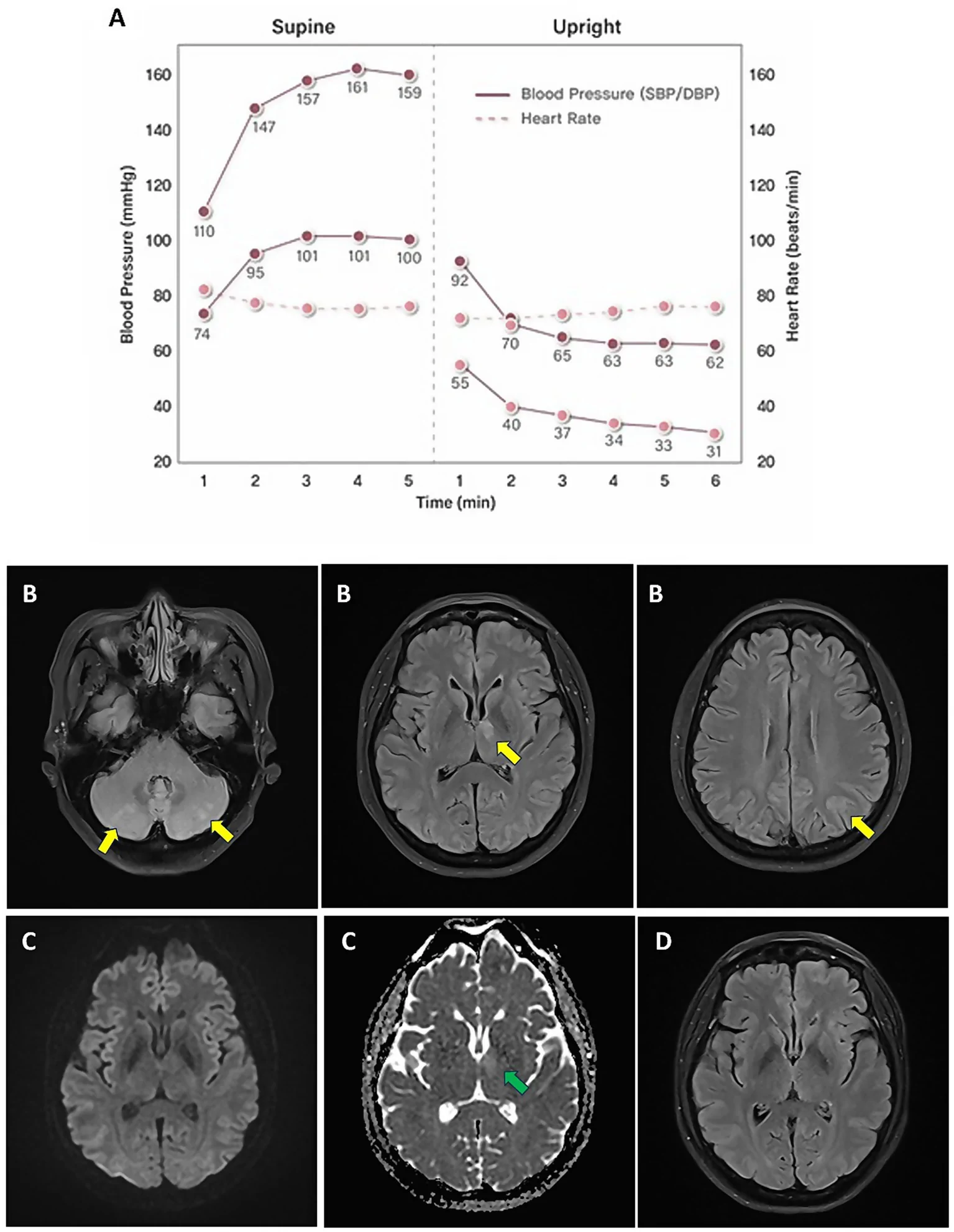
Tilt table test and brain MRI findings. **(A)** During the supine phase, blood pressure was initially within the normal range but gradually increased to the hypertensive range. Upon tilting upright, the patient exhibited a marked drop in systolic and diastolic blood pressure without a compensatory increase in heart rate. The test was terminated at 6 min because of severe dizziness. **(B)** Axial fluid-attenuated inversion recovery (FLAIR) MR image demonstrates multifocal hyperintense lesions involving both cerebellar hemispheres, the left medial thalamus, and the parietal region (yellow arrows). **(C)** Diffusion-weighted imaging (DWI) showed no diffusion restriction, and apparent diffusion coefficient (ADC) maps demonstrated mildly increased signal intensity (green arrow). **(D)** Follow-up FLAIR MR image obtained one week later demonstrated complete resolution of the previously noted lesions.

As orthostatic dizziness persisted for years, the patient presented to our clinic. At our clinic, a tilt-table test showed elevated BP in the supine position, followed by a fall in systolic BP from 159 to 92 mmHg and diastolic BP from 100 to 55 mmHg on head-up tilt without a compensatory heart rate increase (Figure 1A). The patient had demonstrated marked BP lability with preexisting supine hypertension during autonomic testing prior to droxidopa initiation. Valsalva testing demonstrated impaired baroreflex-mediated heart rate and BP responses consistent with severe autonomic failure. Though formal ambulatory BP monitoring data prior to droxidopa initiation were not available, sustained hypertension had not been identified during prior outpatient evaluations. There were no clinical features suggestive of multiple system atrophy, Parkinson disease, peripheral neuropathy, or other neurodegenerative or systemic causes of generalized autonomic failure. Brain MRI was unremarkable. The diagnosis of clinically probable PAF was made based on the presence of severe generalized autonomic failure with neurogenic OH in the absence of parkinsonism or other central neurodegenerative features. Plasma norepinephrine measurement and cutaneous phosphorylated *α*-synuclein immunohistochemistry were not performed because these ancillary investigations were not readily available in our clinical setting. Initial treatment with midodrine and nonpharmacologic measures, including adequate hydration and salt supplementation, did not show meaningful improvement. Midodrine was therefore discontinued, and pyridostigmine together with fludrocortisone was initiated. Because orthostatic symptoms persisted despite partial stabilization of BP, fludrocortisone was discontinued. One month after discontinuation of fludrocortisone, droxidopa therapy was initiated while pyridostigmine was continued at 60 mg twice daily. To minimize adverse effects, droxidopa was titrated as follows: 100 mg once daily for the first 2 days, 100 mg twice daily for the subsequent 2 days, and then 100 mg three times daily thereafter. The last daily dose was administered in the late afternoon to minimize nocturnal supine hypertension. The patient was instructed to avoid prolonged supine positioning, elevate the head of the bed during sleep, and monitor BP during dose escalation. On the fifth day of droxidopa treatment, she developed a mild, diffuse headache accompanied by an elevated BP of 160/90 mmHg, which progressively worsened over the following days. By the 10th day, the headache had intensified to a severe degree, prompting the patient to present to the emergency department. Supine BP was 270/130 mmHg and remained elevated even in the head-up position. Laboratory studies, including complete blood count, liver and renal function tests, electrolytes, lactate dehydrogenase, and urinalysis, were all within normal limits, without evidence of proteinuria or acute kidney injury. Electrocardiography and serum troponin measurements showed no evidence of acute cardiac injury. The patient denied chest pain, and no clinical evidence suggesting secondary endocrine, renovascular, or substance-related causes of hypertensive crisis was identified during the acute evaluation. Despite severe headache and markedly elevated BP, the patient remained fully alert and oriented, without evidence of encephalopathy, seizures, or visual disturbance. Emergency brain MRI revealed multifocal T2-weighted fluid-attenuated inversion recovery (FLAIR) hyperintense lesions involving both cerebellar hemispheres, the left medial thalamus, and the parietal region (Figure 1B). Diffusion-weighted imaging (DWI) showed no diffusion restriction, with mildly increased apparent diffusion coefficient (ADC) values consistent with vasogenic rather than cytotoxic edema (Figure 1C). Gradient-echo imaging (GRE) showed no evidence of hemorrhage, and angiography revealed no significant vascular abnormalities. Although the clinical and radiological features were not fully typical of classic posterior reversible encephalopathy syndrome (PRES), the presence of headache and vasogenic cerebral edema following marked BP elevation suggested hypertensive encephalopathy within the PRES spectrum. In response, droxidopa and pyridostigmine were discontinued, and intravenous nicardipine was promptly administered. Following BP control with intravenous nicardipine, her headache gradually improved. Follow-up MRI demonstrated rapid improvement, with complete resolution of the lesions one week later, accompanied by full remission of the headache (Figure 1D). Pyridostigmine was resumed one week later after discontinuation of droxidopa, and the patient has remained free of recurrent severe hypertension. During the subsequent 12-month follow-up period, no parkinsonism, cerebellar signs, or other central neurodegenerative features suggestive of evolving synucleinopathy emerged.

## Discussion

We described a case of severe hypertension that developed shortly after the initiation of low-dose droxidopa in a patient with PAF. Although supine hypertension is a recognized complication of autonomic failure and droxidopa therapy, the present case highlights that severe hypertensive crisis with reversible vasogenic cerebral edema may occur even during low-dose initiation in vulnerable patients with advanced PAF. PAF is characterized by progressive impairment of baroreflex buffering and widespread sympathetic denervation, leading to neurogenic OH and marked BP lability (1, 3). Although plasma norepinephrine measurement and skin biopsy for phosphorylated *α*-synuclein can provide supportive evidence for PAF, these ancillary investigations were not available in our clinical setting. Therefore, the diagnosis in the present case was based on characteristic clinical manifestations and autonomic function testing findings consistent with clinically probable PAF. Recent longitudinal studies have demonstrated that clinically diagnosed PAF may remain stable or phenoconvert to central *α*-synucleinopathies over time, underscoring the importance of continued clinical follow-up (6). Consistent with these findings, our patient has remained clinically compatible with probable PAF without evidence of phenoconversion during 12 months of follow-up.

In the absence of disease-modifying therapy, management focuses on symptom control through non-pharmacologic strategies and pharmacologic agents to mitigate BP fluctuations (1). Droxidopa, a norepinephrine prodrug, increases norepinephrine availability and thereby enhances vascular tone and standing BP in neurogenic OH (1, 2). Severe BP-related adverse effects associated with droxidopa appear to be rare (2, 5). Although supine hypertension is a recognized complication of droxidopa therapy, reports of hypertensive emergency with reversible vasogenic cerebral edema remain extremely limited in the literature. In advanced PAF, however, impaired baroreflex buffering and autonomic denervation are well-established pathophysiological features that predispose patients to marked BP lability and exaggerated pressor responses to increased norepinephrine availability, thereby increasing susceptibility to severe hypertension, as observed in the present case (7, 8). Denervation supersensitivity has been proposed as a potential mechanism whereby chronically reduced norepinephrine levels may further amplify vascular responses in susceptible individuals (1). Interindividual variability in autonomic denervation and pharmacodynamic responsiveness may also influence susceptibility to severe hypertension during droxidopa treatment.

Pyridostigmine has also been used for neurogenic OH, as it facilitates cholinergic nicotinic neurotransmission at the level of sympathetic ganglia and preferentially augments upright BP without worsening supine hypertension (9). In the present case, pyridostigmine was unlikely to have played a primary role in the development of severe hypertension, as the patient tolerated pyridostigmine both before and after the hypertensive episode without adverse effects. Moreover, pyridostigmine is not known to directly enhance adrenergic neurotransmission or to exacerbate supine hypertension (3, 9). As fludrocortisone had been discontinued one month prior to droxidopa initiation, a residual mineralocorticoid effect is unlikely to have contributed to the hypertensive episode. The patient was also receiving escitalopram, a selective serotonin reuptake inhibitor. To date, there is no published evidence that selective serotonin reuptake inhibitors modify the pressor effects of droxidopa. Given that droxidopa acts via norepinephrine increases outside of the serotonin system, escitalopram was also unlikely to have contributed significantly to this hypertensive crisis.

Although the temporal relationship between droxidopa initiation and the hypertensive crisis supports a plausible association, causality cannot be definitively established based on a single observational case. The patient had underlying severe autonomic dysfunction with preexisting supine hypertension, which may itself predispose to marked BP fluctuations. However, no prior episodes of hypertensive emergency or sustained severe hypertension had been documented before droxidopa initiation. In addition, although concomitant medications were considered unlikely to have played a primary role, potential synergistic or contributing effects cannot be completely excluded. The absence of recurrent severe hypertension after discontinuation of droxidopa, despite resumption of pyridostigmine, further supports droxidopa as a plausible precipitating factor, although causality cannot be definitively established. Pain-related sympathetic activation associated with severe headache may also have contributed to further BP elevation during the acute presentation. Plasma droxidopa or norepinephrine concentrations were not measured during the acute event, limiting pharmacokinetic assessment of the observed adverse response. A formal adverse drug reaction assessment using the Naranjo Adverse Drug Reaction Probability Scale yielded a score of 4, consistent with a possible adverse drug reaction, supporting a plausible but non-definitive association between droxidopa initiation and the hypertensive crisis.

Notably, the patient did not exhibit a typical presentation of PRES. PRES is classically characterized by acute neurological symptoms such as encephalopathy, seizures, or visual disturbances, together with symmetric vasogenic edema predominantly affecting the parieto-occipital regions (10, 11). In contrast, the present case lacked encephalopathy or other typical neurological manifestations of PRES, and the neuroimaging findings were atypical, involving the cerebellum, thalamus, and parietal regions with an asymmetric distribution. Although cerebellar or thalamic involvement has been occasionally reported in PRES, the overall clinical and radiological features in this patient did not fully meet the suggested diagnostic criteria for classic PRES. Taken together, the present case may represent an atypical or incomplete PRES phenotype within the broader spectrum of hypertensive encephalopathy and PRES-related vasogenic edema.

In conclusion, while droxidopa is useful for managing neurogenic OH, clinicians should be aware that severe hypertensive complications with reversible cerebral edema may occur following droxidopa initiation in vulnerable patients with advanced autonomic dysfunction. Baseline assessment of supine BP, gradual dose titration, and home BP monitoring during the early treatment period may help identify excessive pressor responses.

## Data Availability

The raw data supporting the conclusions of this article will be made available by the authors, without undue reservation.
